# Factors involved in the inflammatory events of cervical ripening in humans

**DOI:** 10.1186/1477-7827-2-74

**Published:** 2004-10-22

**Authors:** Ylva Stjernholm-Vladic, Denis Stygar, Christopher Mansson, Britt Masironi, Sonja Akerberg, Hong Wang, Gunvor Ekman-Ordeberg, Lena Sahlin

**Affiliations:** 1 Division for Obstetrics and Gynecology, Department of Woman and Child Health, Karolinska Institutet, Stockholm, Sweden; 2 Division for Reproductive Endocrinology, Department of Woman and Child Health, Karolinska Institutet, Stockholm, Sweden; 3 Present address: Center for Genomics and Bioinformatics, Berzelius vag 35, Karolinska Institutet, Stockholm, Sweden

## Abstract

**Background:**

Cervical ripening is an inflammatory reaction. The glucocorticoid receptor (GR) mediates glucocorticoid anti-inflammatory reactions, whereas nuclear factor (NF)kappaB is a key pro-inflammatory transcription factor. Prostaglandins as well as platelet activating factor (PAF) are inflammatory mediators. Inducible nitric oxide synthase (iNOS) regulates the level of nitric oxide (NO) in response to various inflammatory stimuli. We hypothesize that a changed biological response to glucocorticoids could be a mechanism regulating the inflammatory events resulting in cervical ripening.

**Methods:**

We monitored GR and NFkappaB, prostaglandin synthases cyclooxygenase (COX)-1 and -2, iNOS, as well as the PAF-receptor (PAF-R) in the uterine cervix from term pregnant women (with unripe cervices) before the onset of labor (TP), immediately after parturition (PP), as compared to non-pregnant (NP), using immunohistochemistry and RT-PCR.

**Results:**

The GR protein was detected by immunohistochemistry in the nuclei of stroma and squamous epithelium (SQ). Stromal GR staining was increased in TP as compared to the NP group and decreased again after parturition. GR staining in SQ was decreased after parturition as compared to term. NFkappaB was present in SQ and glandular epithelium (GE), stroma and vascular endothelium. Increased nuclear NFkappaB staining was observed postpartum as compared to term pregnancy in stroma and GE. Stromal immunostaining for COX-1 as well as COX-2 was increased in the TP and PP groups as compared to the NP, and GE displayed an intensely increased COX-2 immunostaining at term and postpartum. Stromal PAF-R immunostaining was highest at term, while it was greatly increased in GE postpartum.

No difference in the immunostaining for iNOS was found between the groups. RT-PCR showed a predominance of GRalpha to GRbeta mRNA in cervical tissue. The COX-2 mRNA level was increased in the PP group as compared to the TP group.

**Conclusions:**

There is a decrease in GR levels in human cervix at parturition. Concomitantly there is an increase of factors such as NFkappaB, PAF-R, COX-1 and COX-2, suggesting that they may participate in the sequence of events leading to the final cervical ripening.

## Background

The human uterine cervix undergoes biochemical changes resulting in softening, effacement and dilatation during pregnancy and labor. This remodeling, or ripening, is a prerequisite for parturition [1]. It is characterized by inflammatory events, such as extravasation of neutrophils and macrophages [2,3] and an increased cervical level of pro-inflammatory cytokines such as interleukin (IL)-8 [3,4]. Progesterone, essential for the maintenance of pregnancy, and glucocorticoids have anti-inflammatory properties [5]. Placental production of progesterone and adrenal synthesis of glucocorticoids [6] increase markedly during human pregnancy. The antiprogestin (RU486), successful for labor induction at term in humans [7], also has anti-glucocorticoid properties. Progesterone and cortisol regulate the human placental corticotropin-releasing hormone (CRH) gene [8]. Placental CRH, synthesized in abundance in the human placental syncytiotrophoblasts and trophoblasts [9], has been proposed to be a key regulator for human parturition trough interactions with adrenal steroids and estrogen [10].

Among the group of structurally related, ligand-inducible nuclear steroid receptor transcription factor proteins, GR was the first that was cloned and sequenced [11]. GR and the progesterone receptor (PR) share structural similarities, and they interact with the same hormone responsive elements [12]. The GRα and GRβ isoforms are derived from the same gene, and GRα is the major form found in human cells and tissues [13].

NFκB is a key pro-inflammatory regulator. GR and NFκB are both inducible transcription factors with diametrically opposed functions in inflammatory responses. A mutual negative direct and indirect cross talk between GR and NFκB has been well described in previous studies [5,14].

Prostaglandin E_2 _(PGE_2_) is widely implicated for cervical ripening in clinical practice [15]. Among the COX enzymes, regulating prostaglandin synthesis, the COX-1 form is constitutively expressed, whereas COX-2 is inducible and particularly involved in inflammatory events [16]. The COX enzymes are down regulated by cortisol in human decidua, myometrium and cervix [17].

Platelet-activating factor (PAF) is a lipid pro-inflammatory mediator, involved in several reproductive processes, i.e. parturition [18]. PAF is synthesized by some leukocytes, blood platelets and vascular endothelial cell [19]. The PAF receptor (PAF-R) is a G-coupled membrane receptor with an estrogen responsive element within its promoter region, enabling regulation by estrogens [20]. The activation of PAF-R is associated with cytoskeletal remodeling and expression of pro-inflammatory modulators, such as COX-2, IL-6 and IL-8 [21]. Thus, PAF-R and COX enzymes have been widely demonstrated as factors involved in the events promoting and proceeding parturition, yet their cell origin in the human uterine cervix remains to be clarified.

Nitric oxide (NO) is synthesized intracellularly from the amino acid L-arginine through the activity of specific synthase enzymes (NOS) [22]. The inducible form, iNOS, present in e.g. macrophages, regulates the level of NO in response to various inflammatory stimuli, including proinflammatory cytokines and lipopolysaccharides.

NO stimulates PGE_2 _release from human cervical tissue explants [23], and is a powerful regulator of COX-2 thereby increasing local PGE_2 _concentrations in inflammatory tissues [24,25]. NO donors do induce cervical ripening in human pregnancy in the first trimester [26,27], at term [28] and in non-pregnant women [29]. Besides, treatment with the NO donor isosorbide-5-mononitrate stimulates production of e.g. COX-2 and PGE_2 _in human cervix [27]. The action of NO on cervical ripening appears to be accomplished by effects on connective tissue and smooth muscle cells in a similar way as previously been shown for prostaglandins [27].

Our hypothesis is that glucocorticoids exert a direct receptor mediated effect in the human cervix uteri, and that a changed biological response to glucocorticoids could be a mechanism behind the events resulting in cervical ripening at parturition. Since NFκB has opposed functions in inflammatory responses as compared to GR, we presume that NFκB could also be a regulator of the inflammatory events leading to cervical ripening. These inflammatory events could be mediated via factors such as the PAF-R, iNOS and/or COX enzymes.

## Methods

### Study patients

All women gave their informed consent and the Local Ethics Committee of the Karolinska Hospital approved the study. All were healthy, had uncomplicated pregnancies and were without medication prior to parturition. The non-pregnant (NP) women were hysterectomised due to benign disorders not involving the cervix. The women in the term pregnant (TP) group all had unripe cervices with a Bishop score <5 points and none of them were in labor. Biopsies were obtained during elective caesarean sections before onset of labor. The biopsies from the post partal (PP) women were taken after a normal vaginal delivery.

For the immunohistochemistry study of GR and NFκB, cervical biopsies were obtained from one para and eleven primipara TP women (n = 12) with a mean age (range) of 33 (28–38) years, and a mean gestational age of 38 (37 to 40) weeks. The women of the PP group (n = 14) were all primipara and had a mean age of 31 (22–37) years, and a gestational age of 40 (39 to 42) weeks. The NP control group (n = 8) had a mean age of 43 (32–49) years, and a mean parity of II (I-III).

The cervical samples available for the immunohistochemistry studies of COX-1, COX-2 and PAF-R were TP (n = 8), PP (n = 10) and NP group (n = 6). Biopsies for RNA preparations were not available for the RT-PCR study from all subjects, NP (n = 5), TP (n = 6) and PP (n = 5). The women in the NP group were significantly older than those of the other two groups. Since hysterectomies in young women are uncommon most biopsies in the NP group are from women in the middle of their 40s, but they were all menstruating regularly and did not receive any medication.

### Tissue collection

Cervical biopsies were obtained transvaginally (for the TP and PP groups) from the anterior cervical lip at the 12 o'clock position, from 10–20 mm depth. The tissue samples from the hysterectomies were obtained directly after the uterus was removed during operation. The same physician (YSV) collected all the samples. The biopsies were immersion-fixed in 4% phosphate buffered formaldehyde at 4°C overnight, stored at 4°C in 70% ethanol and thereafter embedded in paraffin. From the biopsies that were large enough, a small piece was cut off prior to fixation, and frozen in -70°C until RNA preparation.

### RNA preparation and reverse transcription

Total RNA from frozen cervical tissue samples was purified with the SV Total RNA isolation system (Promega, Madison, WI) according to a procedure recommended by manufacturer. One microgram of total RNA from each sample was reverse transcribed at 42°C for 45 min in a final volume of 40 μl with a reaction mixture containing 50 mmol/l Tris-HCl (pH 8.3), 75 mmol/l KCl, 3 mmol/l MgCl_2_, 7.5 mmol/l dithiothretiol, 0.5 mmol dNTPs, 1 μg random hexameters, and 400 U of Moloney murine leukemia virus reverse transcriptase (Gibco-BRL, Paisley, UK).

### RT-PCR

Oligonucleotide primers for the GRα gene were as follows [30]: 5'-CCT AAG GAC GGT CTG AAG AGC-3' and 5'-GCC AAG TCT TGG CCC TCT AT-3', corresponding to nucleotides 2158-2178 and 2616-2635 of the human GRα cDNA (GenBank accession No X03225). Oligonucleotide primers for the GRβ gene were as follows [30]: 5'-CCT AAG GAC GGT CTG AAG AGC-3' and 5'-CCA CGT ATC CTA AAA GGG CAC-3', corresponding to nucleotides 2158-2178 and 2503-2523 of the human GRβ cDNA (GenBank accession No X03348). Oligonucleotide primers for the COX-1 gene were as follows: 5'-TGC CCA GCT CCT GGC CCG CCG CTT-3' and 5'-GTG CAT CAA CAC AGG CGC CTC TTC-3', corresponding to nucleotides 568-591 and 871-847 of the human COX-1 cDNA [31]. Oligonucleotide primers for the COX-2 gene were as follows: 5'-TTC AAA TGA GAT TGT GGG AAA ATT GCT-3' and 5'-AGA TCA TCT CTG CCT GAG TAT CTT-3', corresponding to nucleotides 574-601 and 878-854 of the human COX-2 cDNA [32]. The predicted size of the PCR products was 477 bp for GRα, 366 bp for GRβ, 304 bp for COX-1 and 305 bp for COX-2.

For PCR, the cDNAs corresponding to 50 ng RNA were added to 10 μl of HotStarTaq^® ^master mix (Quiagen GmbH, Hilden, Germany) containing 2.5 μM of each oligonucleotide primer in a final volume of 20 μl. The reaction mixture was overlaid with mineral oil. After an initial incubation for 15 min at 95°C, the samples were subjected to 33 (GRα, COX-1 and COX-2) and 40 (GRβ) cycles of 30 s at 94°C, 40 s at 60°C, and 60 s at 72°C with a final extension step at 72°C for 10 min in the DNA Thermal Cycler 480 (Perkin-Elmer, Norwalk, CT). The amount of PCR product for GRα increased linearly up to 36 cycles and for COX-1 and COX-2 it increased linearly up to 38 cycles (data not shown). Quantitative measurement of GRβ mRNA would require larger amounts of cervical RNA than were available, since GRβ showed very low expression with a visible band only after 40 cycles.

To standardize the quantification method, an endogenous 18S rRNA was used as an internal standard. The 18S rRNA primers and Competimers™ (modified at their 3' ends to block extension by DNA polymerase) were obtained from Ambion (Quantum RNA 18S Internal Standards; Ambion Austin, TX). The standard, and the GR and COX mRNAs were amplified in parallel and under the same conditions. A mixture of 18S primers and Competimers™ (1:9) was used to modulate amplification efficiency of 18S rRNA to the same linear range as GRα, COX-1 and COX-2 when amplified under the same conditions. The predicted size of the PCR product for 18S was 489 bp. The PCR products were run on 2% agarose gel and stained with ethidium bromide. Bands were captured and analyzed using ChemiDoc Gel Documentation System (Bio-Rad Laboratories, Hercules, CA). The levels of GR and COX PCR products were normalized against the 18S product. The RT-PCRs were repeated twice.

### Immunohistochemistry

Paraffin sections (5 μm) were used and a standard immunohistochemical technique (avidin-biotin-peroxidase) was carried out as described before [33] to visualize GR, NFκB, COX-1, COX-2, PAF-R and iNOS. After the tissues were dewaxed and rehydrated, an antigen retrieval procedure was performed. The sections were pre-treated by heating in a microwave oven at 700 W in 0.01 M sodium citrate buffer (pH 6.0). Endogenous peroxidase activity was blocked by incubation with 3% hydrogen peroxide. All tissue sections were exposed to a non-immune block with normal goat serum. A polyclonal rabbit anti-human antibody was used for the detection of GR (ABR PAI-511A, Affinity Bioreagents, Inc., Golden, CO, USA). This antibody recognizes both GRα and GRβ. The tissue sections were incubated over night in +4°C with the primary antibody diluted 1:1000. A polyclonal rabbit anti-human antibody (DB033 Delta Biolabs, Cambell, CA, USA) was used for the NFκB immunostaining. The tissue sections were incubated with the primary antibody diluted 1:500 over night at +4°C. The primary antibodies were replaced with non-immune rabbit IgG in negative controls. Polyclonal goat anti-human (sc-1752 and sc-1745, Santa Cruz) antibodies were used (diluted 1:100) for the COX-1 and COX-2 respectively. The incubation with primary antibody was 1 hr at 37°C for both COX-1 and COX-2. A polyclonal goat anti-human (sc-8741, Santa Cruz) antibody was used for the PAF-R immunostaining. The tissue sections were incubated with the primary antibody diluted 1:100 over night at +4°C.

Replacing the primary antibody with non-immune goat IgG was used as negative control.

A monoclonal mouse anti-iNOS antibody was used for detection of iNOS (N32020, Transduction Laboratories, Lexington, KY, USA). It recognizes the C-terminal domain of iNOS. The antibody was diluted 1:50 and incubated for 70 minutes in room temperature. Replacing the primary antibody with non-immune mouse IgG was used as negative control.

### Image analysis

A microscope and CCD video camera connected to a computer with an image analysis program (Leica Imaging System Ltd., Cambridge, UK) was used to assess quantitative values from GR and NFκB immunohistochemistry. The quantification of nuclear immunostaining was performed on the digitized images of systematic randomly selected fields of stroma and squamous epithelium. Ten fields were analyzed separately in each section of tissue, using the color-discrimination software. Positive staining is presented as a ratio of the area of positively stained nuclei (brown) to the total area of cell nuclei (brown and blue).

### Manual scoring

Two observers blinded to the identity of the slides, performed all the assessments. The staining was evaluated semi-quantitatively using a grading system. The staining intensity was graded on a scale of (0) no staining, (1) very faint, (2) faint, (3) moderate and (4) strong staining.

### Statistics

Statistical calculations for the data from the relative quantification of RT-PCR products, the immunohistochemistry results by image analysis and manual scoring were performed by ANOVA on Ranks (Kruskal-Wallis' test) and significances were evaluated by Dunn's test. Values with different letter designations are significantly different (p < 0.05).

## Results

### GR

**Figure 1:**
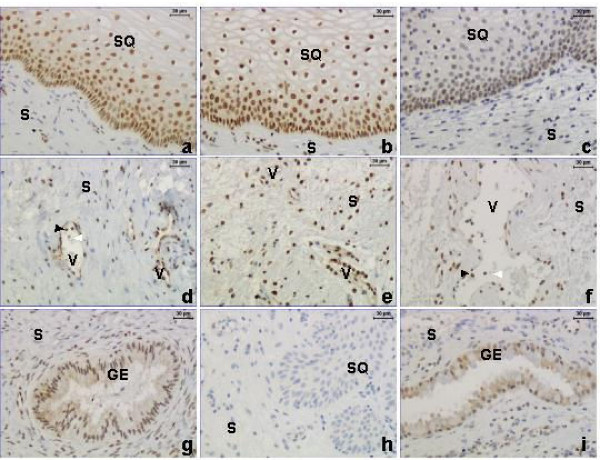
Immunostaining of GR in the non-pregnant (NP) (left column, **a**, **d**, **g**), term pregnant (TP) (middle column, **b**, **e**, **h**) and postpartum (PP) (right column, **c**, **f**, **i**) groups. GR protein (brown staining) is present in the nuclei of cervical stroma (S), squamous epithelium (SQ), vessels (V) and glandular epithelium (GE) in all three groups. Some leukocytes, identified by their morphology, were positive for GR (black arrowheads) while others were negative (white arrowheads) (**d **and **f**). A negative control where the primary antibody was replaced by rabbit IgG is shown in **h**.

**Figure 2:**
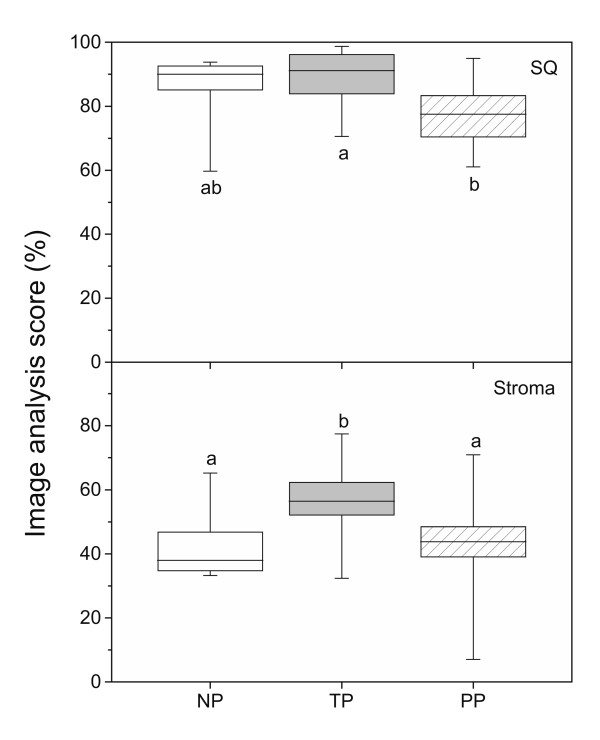
GR levels, as assessed by image analysis of immunohistochemistry results, in cervical squamous epithelium (SQ) and stroma in samples from TP (n = 12) and PP (n = 14) as compared to NP (n = 8) women. Box and whisker plots represent the median value with 50% of all data falling within the box. The "whiskers" extend to the 5^th ^and 95^th ^percentiles. Boxes with different letter designations are significantly different, p < 0.05.

By immunohistochemistry the GR protein was localized to the nuclei of cervical stroma (S), squamous epithelium (SQ), glandular epithelium (GE) and vascular endothelium (Figure 1a,1b,1c,1d,1e,1f,1g,1h,1i) in samples from the NP (left column), TP (middle column) and PP (right column) groups. By image analysis the GR levels were determined in SQ and stroma (Figure 2). The stromal cells include vascular epithelium and the leukocytes within the stroma. Blood cells within vessels (V) are excluded from the image analysis. Strong immunostaining was present in SQ, particularly in the basal and parabasal cell layers (Figure 1a,1b,1c). There was a significant decrease in immunostaining of the PP group as compared to the TP group, both in stroma and SQ (Figure 2). The stromal GR immunostaining was increased in the TP group as compared to the NP and PP groups (Figure 2). It was noted in all groups that GE (Figure 1g and 1i), vascular endothelium (Figure 1d,1e and 1f) and some intravascular and perivascular leukocytes (as identified by their morphology, black arrowheads, Figure 1d and 1f) stained positive for GR, while some leukocytes were negative (white arrowheads, Figure 1d and 1f).

### NFκB

**Figure 3:**
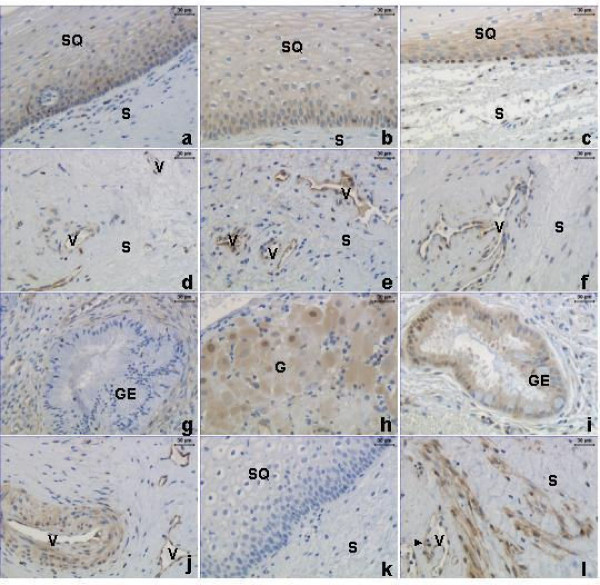
Immunostaining of NFκB protein in stroma (S), glandular epithelium (GE), squamous epithelium (SQ) and vessels (V) in cervical biopsies from the NP (left column, **a**, **d**, **g**, **j**), TP (middle column, **b**, **e**, **h**) and PP (right column, **c**, **f**, **i**, **l**) groups. Positive nuclear and cytoplasmic immunostaining is also observed in neuronal ganglions (G) (**h**). Some leukocytes, identified by their morphology, display positive NFκB staining (**l**, black arrowhead). A negative control where the primary antibody is replaced by rabbit IgG is shown in **k**.

**Figure 4:**
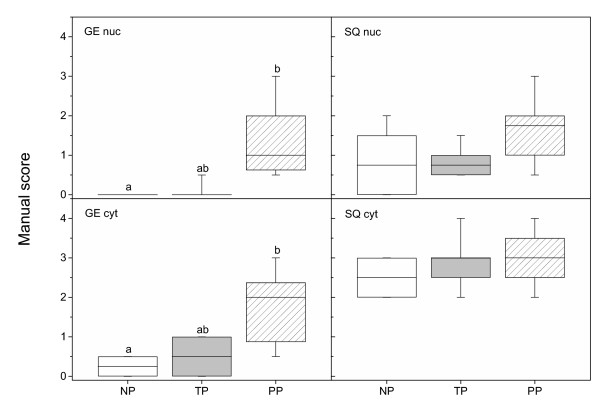
NFκB protein levels, as assessed by manual scoring of immunohistochemistry results, in nuclei and cytoplasma of glandular epithelium (GE) and squamous epithelium (SQ) in cervical samples from TP (n = 10) and PP (n = 10) as compared to NP (n = 8) women. Box and whisker plots represent the median value with 50% of all data falling within the box. The "whiskers" extend to the 5^th ^and 95^th ^percentiles. Boxes with different letter designations are significantly different, p < 0.05.

**Figure 5:**
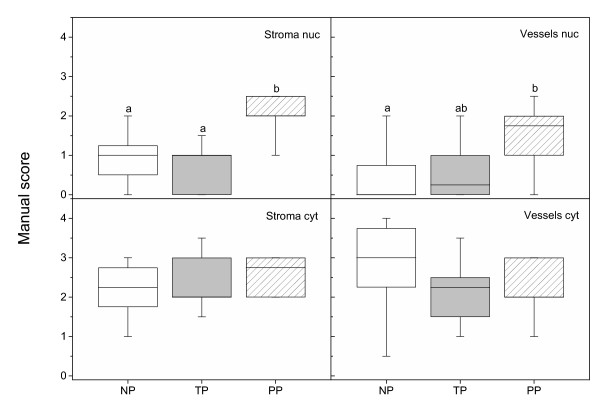
NFκB protein levels, as assessed by manual scoring, in nuclei and cytoplasma of stroma and vascular endothelium in cervical samples from TP (n = 10) and PP (n = 10) as compared to NP (n = 8) women. Box and whisker plots represent the median value with 50% of all data falling within the box. The "whiskers" extend to the 5^th ^and 95^th ^percentiles. Boxes with different letter designations are significantly different, p < 0.05.

Positive NFκB immunostaining in cervix uteri was present in stroma, GE, SQ and vascular endothelium in NP (left column), TP (middle column) and PP (right column) groups (Figure 3a,3b,3c,3d,3e,3f,3g,3h,3i,3j,3k,3l). Strong positive staining for NFκB was also seen in neuronal ganglions (G, Figure 3h) and in smooth muscle cells/activated fibroblasts, both around blood vessels (Figure 3j) and within the stroma (Figure 3l). In GE the nuclear and cytoplasmatic NFκB immunostaining was increased in the PP group as compared to the NP group (Figure 4). No changes in NFκB staining were observed in SQ (Figure 4). The stroma displayed an increased nuclear immunostaining, but unchanged cytoplasmatic staining, in the PP group as compared to the other groups (Figure 5). Vascular endothelium showed an increased nuclear but unchanged cytoplasmatic staining, in the PP group as compared to the NP group (Figure 5). In all groups some leukocytes (black arrowhead, Figure 3l), stained positive for NFκB. As for image analysis of GR, the manual scoring of NFκB in stromal cells could include leukocytes within the stroma, but not blood cells within vessels.

### COX-1

**Figure 6:**
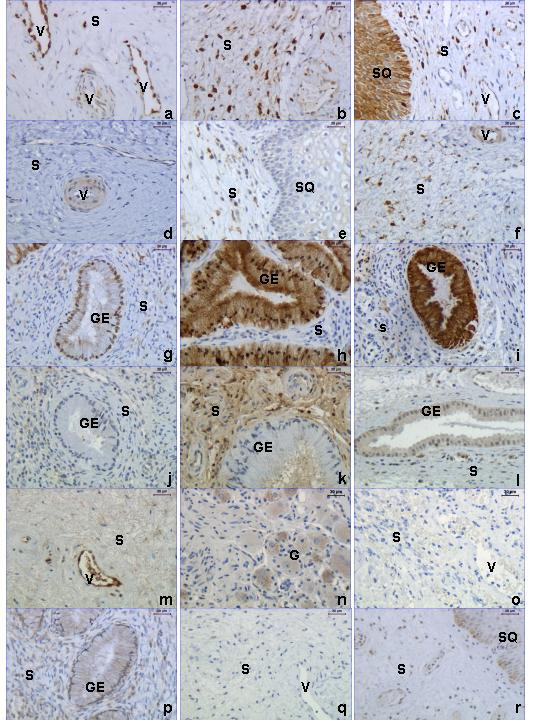
Immunostaining of COX-1 (**a-c**), COX-2 (**d-i**), PAF-R (**j-n**) and iNOS (**p**, **r**), in cervices from women in the NP (left column), TP (middle column) and PP (right column) group. A representative negative control for COX-1, COX-2 and PAF-R immunohistochemistry (primary antibody replaced by goat IgG, same secondary antibody) is shown in **o**, the negative control for iNOS is shown in **q **(primary antibody replaced by mouse IgG). Abbreviations: S = stroma, GE = glandular epithelium, SQ = squamous epithelium, V = vessel and G = ganglion cells.

**Figure 7:**
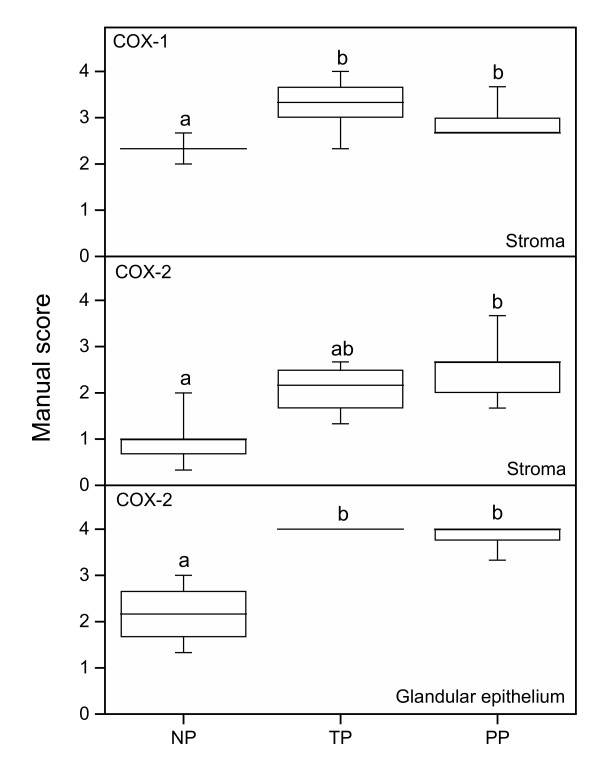
COX-1 (top) and COX-2 (middle, bottom) protein levels, as assessed by manual scoring of stroma (top, middle) and glandular epithelium (bottom) in cervical samples from the TP (n = 8), PP (n = 9) and NP (n = 6) groups. Box and whisker plots represent the median value with 50% of all data falling within the box. The "whiskers" extend to the 5^th ^and 95^th ^percentiles. Boxes with different letter designations are significantly different, p < 0.05.

Immunostaining for COX-1 (Figure 6a,6b,6c) was found in platelets (not shown), some leukocytes (not shown), vessel endothelium (Figure 6a,6c), stroma (Figure 6a,6b,6c), neuronal ganglion (not shown), SQ (Figure 6c) and GE (not shown). Manual scoring was performed and there was a significant increase of COX-1 staining in the stroma of the TP and PP groups as compared to the NP group (Figure 7, top). No differences were found in staining of the SQ, GE and endothelium between the three groups (data not shown).

### COX-2

Immunostaining of COX-2 (Figure 6d,6e,6f,6g,6h,6i) was found in the stroma, GE and smooth muscle cells/activated fibroblasts, both around vessels and arranged as bundles within the stroma. The intensity of COX-2 staining was overall less than that of COX-1, except in GE where the immunostaining was almost maximal in all samples of the TP and PP groups (Figure 6h,6i). There was an increased immunostaining in the stroma of the PP group and in GE from the TP and PP groups, as compared to the NP group (Figure 7 middle and bottom, respectively).

### PAF-R

**Figure 8:**
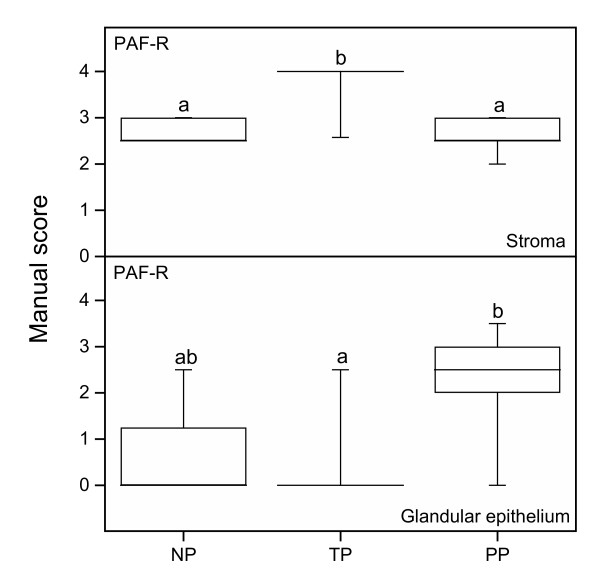
PAF-R protein levels, as assessed by manual scoring, in stroma (top) and glandular epithelium (bottom) in cervical samples from the TP (n = 11), PP (n = 13) and NP (n = 9) groups. Box and whisker plots represent the median value with 50% of all data falling within the box. The "whiskers" extend to the 5^th ^and 95^th ^percentiles. Boxes with different letter designations are significantly different, p < 0.05.

Immunostaining of PAF-R was found in stroma, neuronal ganglion (G) and GE (Figure 6j,6k,6l,6m,6n). The immunostaining in the stroma was higher in the TP group (Figure 6k) compared with NP (Figure 6j) and PP groups (Figure 6l) (Figure 8 top). The immunostaining in GE was increased in the PP group (Figure 6l) as compared to the TP group (Figure 6k) (Figure 8 bottom). A negative control representative for the goat-derived antibodies, where goat IgG replaced the primary antibody, is shown in Figure 6o.

### iNOS

Faint positive staining was found in SQ, stroma and GE (Figure 6p,6r). There were no differences in immunostaining of iNOS found between the groups (data not shown). A negative control where the primary antibody was replaced by mouse IgG is shown in Figure 6q.

### GRα mRNA

**Figure 9:**
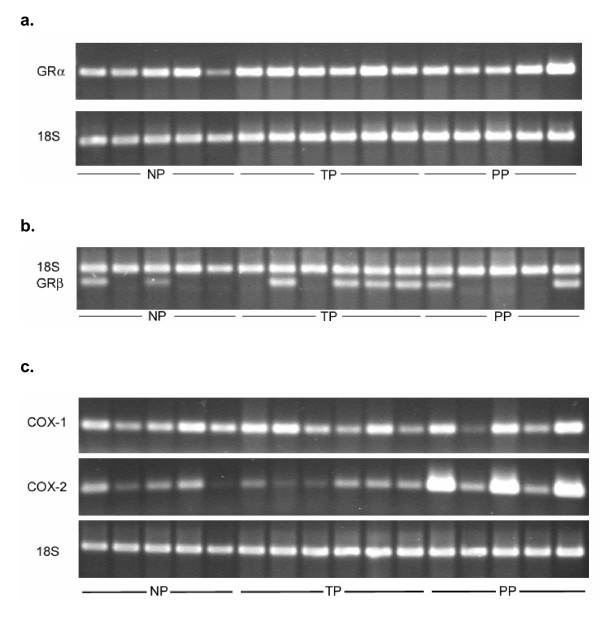
Images of representative RT-PCR gels for **a**: GRα, **b**: GRβ and **c**: COX-1 (upper band) and COX-2 (middle band) in the human cervix in the non-pregnant (NP; n = 5), term pregnant (TP; n = 6) and postpartum (PP; n = 5) groups. The gels are stained with ethidium bromide. **a**: GRαmRNA(upper band) and 18S mRNA (lower band). **b**: 18S mRNA (upper band) and GRβmRNA(lower band). **c**: The 18S mRNA (bottom band) and the COX-1 (upper band) and COX-2 (middle band) PCR products.

The GRα mRNA level was determined by RT-PCR (Figure 9). GRα mRNA levels were not significantly different between the three study groups, but there was a tendency of an increased level in the TP group as compared to the other two groups (Figure 9a). When the relative GRα mRNA level in the NP group was defined to 100%, the TP group showed 120% and the PP group 90% of that level.

### GRβ mRNA

Very low levels of GRβ mRNA were present in 4 out of 6 samples from the TP group, and in 2 out of 5 samples in the NP and PP groups (Figure 9b).

### COX-1 mRNA

**Figure 10:**
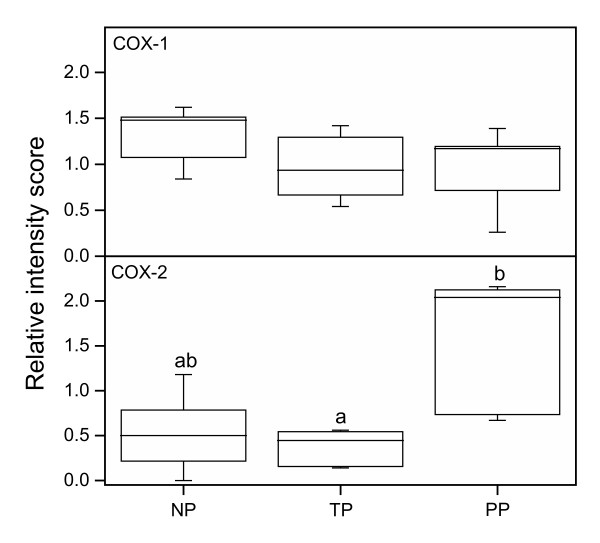
COX-1 (top) and COX-2 (bottom) mRNA levels in the human cervix from the NP (n = 5), the TP (n = 6) and the PP (n = 5) groups as determined by RT-PCR. Intensities of the PCR product bands were normalized against the internal 18S standard. Box and whisker plots represent the median value with 50% of all data falling within the box. The whiskers extend to the 5th and 95th percentiles. Group medians with different letter superscripts are significantly different (P < 0.05).

There was no difference in the COX-1 mRNA level between the groups, as assessed by RT-PCR (Figure 9c) (Figure 10, top).

### COX-2 mRNA

The COX-2 mRNA level was the highest in the PP group (Figure 9c) (Figure 10, bottom), but there was a great variation between the individual PP patients (Figure 9c), probably due to the different amount of glands present in the biopsies.

## Discussion

This study is, to our knowledge, the first reporting GR expression in human cervix uteri during pregnancy and at parturition. In a recent study on human endometrium [34], GR protein was observed in stromal cells and within leukocytes invading the stroma. Pujols et al. [13] reported that the expression of GRα mRNA is 400-fold higher than GRβ mRNA expression in human tissues. The GRα protein was found in all cells and specimens in their study, while GRβ was not detected in any specimen. In our study, GRα mRNA was much more abundant than GRβ mRNA. Therefore, we conclude that GRα is the major receptor type observed in our study, although GRβ could contribute to the GR protein level in the TP group.

Glucocorticoids exert their anti-inflammatory effects primarily by inhibiting the expression of cytokines e.g. IL-8, as demonstrated in human fibroblasts [35], tumour necrosis factor (TNF)-α [36], colony-stimulating factor (CSF) and macrophage stimulating factor (M-CSF) [5]. The majority of these pro-inflammatory genes have no glucocorticoid responsive elements (GRE) in their promoter regions that could explain the effect of glucocorticoids, but many of them contain sites for the transcription factors activating protein (AP)-1 and NFκB [37]. In the review by McKay and Cidlowski [[5], and references therein] it is obvious though, after comparing the genes transcriptionally regulated by NFκB (e.g. IL-8, iNOS, COX-2) and genes repressed by GR (e.g. IL-8, iNOS, COX-2, GR, CRH, fibronectin, and metalloproteinases (MMPs)), that NFκB and GR have diametrically opposed functions. The oppositely regulated IL-8, iNOS and COX-2 have all proven to be important for cervical ripening and labor induction [4,17,38].

Maternal plasma levels of cortisol increase until term [6], but their diurnal variation is maintained [39] although more long-term variations have been suggested [40]. At parturition, maternal plasma levels of placental CRH [10,41], pituitary adrenocorticotropic hormone (ACTH) and adrenal cortisol [6] increase exponentially.

Our data shows that the GR protein was present in cervical stroma, SQ and vascular endothelium in samples from non-pregnant, term pregnant and postpartum women. The decrease in GR levels in stroma and SQ at parturition, as compared to term, could be interpreted in terms of a GR mediated glucocorticoid anti-inflammatory activity during pregnancy that is ended at parturition.

The NFκB protein was present in cervices from non-pregnant, term pregnant and postpartum women. This protein, ubiquitously expressed in a variety of cell types, is found in the cytoplasm in its inactive form, but translocates into the nucleus upon activation [14]. Therefore, the increase in nuclear NFκB levels in cervical stroma, GE and vascular endothelium in the PP group suggests an activation of NFκB at parturition. Thus, our present results of decreased GR and increased NFκB levels at parturition are in agreement with the reports of their opposed effects on the activity of several inflammatory genes [[5], and references therein] and the idea of cervical ripening being an inflammatory reaction [42].

Polymorphonuclear leukocytes and macrophages migrate from blood vessels and accumulate in the cervix uteri before parturition [2]. GR and NFκB were identified by immunohistochemistry in morphologically recognized leukocytes in the samples from all groups in this study. GR-positive leukocytes have also been observed in the endometrium of non-pregnant women and the decidua of early pregnant women [43].

Cortisol is a potent inhibitor of COX-2 in myometrium, decidua and cervix [[17], and references therein]. We found the constitutive COX-1 protein to be present at higher levels in the stroma at term and postpartum, whereas COX-1 mRNA was unchanged. Inducible COX-2 mRNA was increased at parturition as compared to term, and the COX-2 protein was increased in stroma postpartum and in GE at term and postpartum as compared to the NP group. Our observations suggest, that prostaglandin synthesis occur especially in the GE, where a highly intensive COX-2 immunostaining was observed at term and postpartum. This is in agreement with a recent report on COX-2 mRNA in the pregnant baboon cervix [44]. This would also explain the large variation in COX-2 mRNA levels found in the present study. The RNA is prepared from homogenates of cervical biopsies, and the amount of glands present in the biopsies varies from none to plenty. Our results indicate that prostaglandin synthesis could be regulated predominantly via COX-2 in cervical GE at parturition. The stromal increase in COX enzymes could be due to the influx of macrophages and leukocytes before parturition [2], since leukocytes are an abundant source of PGE_2 _in the human body [45]. We could not exclude that COX-1 and COX-2 are present in the stromal cells, thereby adding another cervical source of prostaglandin synthesis, but it seems likely that the enzymes are mainly expressed in the invading leukocytes. Further, our data together with previous findings, suggest suppression not only by progesterone [46], but also by cortisol [[17], and references therein] of prostaglandin synthesis in the human uterine cervix during pregnancy and a release of this suppression at parturition. Since the COX-2 promoter contains NFκB binding sites [47], the activation of COX-2 could be regulated via NFκB.

Nitric oxide stimulates PGE_2 _release from human cervical tissue explants [23]. Nitric oxide donors induce cervical ripening in term pregnancy in humans [28]. Treatment with the NO donor isosorbid-5-mononitrate stimulates the synthesis of COX-2 and PGE_2 _in human uterine cervix [27]. The immunostaining of iNOS in the present study did not differ between the groups, and thus did not vary due to pregnancy or parturition. This is not in agreement with previous studies on human cervix [38,48]. Tschugguel et al. found increased iNOS immunostaining with an antibody from Transduction laboratories (no number stated), but not on the mRNA level using RT-PCR, indicating a post-transcriptional regulation of iNOS [38]. Ledingham et al. [48] found increased cervical immunostaining and stronger bands on Western blot in term pregnancy as compared to the non-pregnant state, using an antibody from Transduction laboratories (39120, clone 54). We also used a monoclonal iNOS antibody from Transduction laboratories, but a different clone (32020, clone 6), which could explain the different results. We found some staining in GE, while Ledingham et al. state no staining of the glands [48].

Platelet activating factor (PAF), like prostaglandins derived from the arachidonic acid precursor, is a multifactorial pro-inflammatory mediator, which has been implicated in parturition. Local application of PAF in rats induced cervical ripening [49], whereas a PAF-R antagonist prolonged parturition [50]. PAF-R has been identified in human cervical fibroblasts in vitro [51]. We show, for the first time, presence of the PAF-R protein in the human uterine cervix in vivo. Stromal PAF-R immunostaining was most pronounced at term, and decreased after parturition. PAF-R immunostaining was, like for COX-2, further increased in GE postpartum. PAF increases the expression of pro-inflammatory cytokines e.g. IL-8, and this effect can be abolished using a PAF-R antagonist (WEB2170) [18,51]. Furthermore, the COX-2 promoter contains NFκB binding sites [47], and the PAF stimulated COX-2 induction is NFκB dependent [51], indicating that the PAF-R could activate NFκB and thereby induce COX-2. PAF also increases expression of MMP-1 [51], which has been shown to effectuate collagen degradation and cervical ripening [1,52].

If the process of cervical ripening is disturbed, either resulting in a preterm delivery or to a prolonged delivery time, possibly ended by a cesarean section, it will lead to increased risks for both the mother and the child. Preterm delivery is the leading factor causing neonatal mortality and morbidity [54]. An increased knowledge of the factors regulating the cervical ripening process will give tools for developing pharmaceuticals that can regulate cervical ripening.

## Conclusions

We have demonstrated that the human uterine cervix is a potential target organ for glucocorticoids during pregnancy. The higher GR protein levels in cervical stroma and SQ before parturition may reflect a GR mediated anti-inflammatory effect of cortisol during pregnancy, with a subsequent decline of this activity at parturition. The concomitant increase in nuclear NFκB levels in the cervix suggests activation of this transcription factor at parturition. NFκB activity promotes pro-inflammatory events and could be responsible, at least in part, for the observed increase in COX-1, COX-2 and PAF-R levels.
